# The Influence of Different Distance Sampling Step Sizes on the Accuracy of Neural Network-Based Magnetic Distance Measurement

**DOI:** 10.3390/s26206583

**Published:** 2026-10-16

**Authors:** Shihan Wang, Qiao Qi, Chaoyi Qiu, Huachen Zhu, Zhe Sheng, Xiumei Li, Ru Bai, Zhenghong Qian

**Affiliations:** 1 School of Information Science and Technology, Hangzhou Normal University, Hangzhou 311121, China; 2024112011009@stu.hznu.edu.cn (S.W.); qiqiao@hznu.edu.cn (Q.Q.); cy.qiu@joysonquin.com (C.Q.); zhesheng@hznu.edu.cn (Z.S.); xiumei_li@hotmail.com (X.L.); bairu@hdu.edu.cn (R.B.); 2 Joysonquin Automotive Systems, Ningbo 315040, China; hc.zhu@joysonquin.com; 3 Center for Integrated Spintronic Devices, Hangzhou Dianzi University, Hangzhou 310018, China

**Keywords:** neural networks, magnetic sensors, distance measurement, sampling step size

## Abstract

Achieving high accuracy in neural network-based magnetic distance measurement depends critically on the spatial sampling step sizes of the training data and the number of deployed sensors. This paper systematically investigates how these two distinct factors influence the prediction accuracy of deep neural networks (DNNs). Utilizing Ansys finite element simulations of a permanent magnet translating over a 140 mm range, it is found that finer sampling steps drastically improve DNN precision. Specifically, decreasing the sampling step from 0.5 mm to 0.005 mm for a single-sensor system reduces the mean absolute error (MAE) from 0.9405 mm to 0.0503 mm. Furthermore, expanding the setup to a three-sensor array under identical conditions further suppresses the simulated MAE from 0.2407 mm to 0.0016 mm. Empirical experiments subsequently validated these theoretical findings. Notably, the physical three-sensor setup achieved a minimum MAE of 1.1881 mm at the finest step size, which represents a marked improvement over the 1.6065 mm error observed in the single-sensor system. These results confirm that both a sufficient sample size and multiple sensors enhance distance measurement accuracy.

## 1. Introduction

Magnetic sensors serve as an indispensable tool for acquiring the spatial information of magnetic sources by detecting magnetic field distributions. Due to their non-contact nature, high precision, and environmental robustness, they are widely used in non-contact sensing, position control, and high-precision operations [1,2,3,4,5,6].

Current methodologies for magnetic distance measurement can be primarily categorized into traditional technical methods and neural network (NN)-based methods. Traditional technical methods have historically provided measurement capabilities and have reached high levels of performance, frequently through the implementation of sensor redundancy. For example, Sherman et al. [7] reported a maximum error of 1.8 mm in their experiments, which collected data from 27 sensors spaced 1 mm apart for a magnet moving axially within a 100 mm range. Schlageter et al. [8] utilized sixteen Hall sensors arranged in a 4 × 4 matrix and reported an average tracking error of 3 mm at a testing distance of 140 mm. Taghvaeeyan and Rajamani [9] achieved a maximum position error of less than 0.4 mm within a 50 mm displacement and reduced positioning error to under 1 mm within 47 ms after interference by employing redundant magnetic sensors.

Despite the established efficacy of traditional techniques, NN-based methods have increasingly become prominent due to their significant advantages in non-linear mapping, adaptive feature extraction, and the learning of complex spatial patterns [10,11,12,13]. These characteristics make it highly feasible to establish a precise mapping from raw magnetic signals directly to target distances [14,15]. Consequently, diverse architectures, including backpropagation (BP) NNs [16], multilayer perceptron (MLP) [17,18], convolutional neural networks (CNNs) [19,20,21], and long short-term memory (LSTM) [22], have been applied to research in magnetic positioning and distance measurement. Within the domain of NN-based methods, significant contributions have been reported. For instance, Chen et al. [23] trained a network using a dataset from magnetic target simulations and reported an average displacement error of 2.15 cm and a maximum error of 5.20 cm over a movement range of 61–79 cm. Similarly, Lv et al. [16] trained a neural network using 9 sensors and 1110 sampling points, achieving an average position error of 8.95 ± 1.74 mm over a 216–296 mm range. However, their study also indicated that extending the tracking range to 396 mm increased the error to 16.68 ± 6.25 mm. In contrast, recent studies have further improved the positioning accuracy by introducing more advanced network structures or learning strategies. For example, Su et al. [24] presented the few-shot learning network AMagPoseNet with a localization error of 1.87 ± 1.14 mm for magnet pose estimation. Wang et al. [25] adopted transfer learning and four three-channel sensors, reaching a mean position error of 1.66 mm in a 100 × 100 × 100 mm workspace for medical positioning tasks.

While these advancements have been made, the existing literature has not adequately addressed the correlation between measurement performance and spatial sampling resolution, specifically the distance sampling step size. In our research group, Qiu et al. [26] demonstrated the application of NNs in measuring distance with magnetic sensors and noted that using more sensors could further enhance measurement precision. Building on this foundation, this paper introduces a comprehensive study on the impact of distance sampling step sizes when employing deep neural networks (DNNs) for magnetic distance measurement. Specifically, we systematically analyze the effect of varying sampling resolutions on DNN training efficacy and validate these theoretical findings using magnetic field data acquired from a physical experimental platform.

The rest of this paper is structured as follows: Section 2 presents the fundamental principles and architecture of the DNNs. Next, Section 3 describes the simulation results for training the neural network and analyzes the impact of distance sampling step size on training performance. Then, in Section 4, magnetic field data from a physical experimental platform are collected by moving a permanent magnet and then input into the trained network for distance prediction. Finally, Section 5 summarizes and discusses the DNN’s predictive performance in distance measurement.

## 2. DNN Principles and Architecture for Distance Measurement

NNs, characterized by their nonlinear fitting and feature learning capabilities, are uniquely suited for extracting complex mapping relationships directly from raw magnetic field data. By deploying NNs to magnetic ranging tasks, a robust nonlinear mapping from magnetic field signals to target distance can be established [27]. In such an architecture, magnetic field data enter through the input layer and undergo sequential nonlinear transformations across multiple hidden layers. Within these layers, neurons process signals using activation functions to ultimately produce a distance estimate at the output layer. During the training phase, backpropagation and gradient descent algorithms are employed to optimize model performance by minimizing the error between predicted distances and ground-truth values through iterative weight and bias adjustments.

While foundational neural networks provide the basic mechanism for data mapping, increasing the depth of these structures leads to the formation of DNNs, which are specifically designed to handle the high-dimensional nonlinearities inherent in magnetic sensing. Such DNN architectures offer distinct technical advantages for distance measurement using magnetic sensors. First, while the magnetic field intensity of a permanent magnet follows complex nonlinear laws with varying distance, DNNs can effectively model these patterns. Traditional physical modeling methods are often vulnerable to environmental interference, such as metallic shielding and geomagnetic noise, or suffer from model simplification errors. In contrast, DNNs automatically extract interference-resistant, distance-related features from magnetic field data, eliminating the need for precise physical models and thereby enhancing measurement robustness and generalization. Furthermore, the validated performance of DNNs in complex scenarios, such as magnetic positioning [28], provides a reliable technical foundation for their adaptation to high-precision distance measurement tasks.

In this study, the construction of a specific DNN architecture is tailored for distance measurement. The network consists of one input layer, three hidden layers, and one output layer. The number of neurons in the three hidden layers is set to 20, 25, and 30. For model training, all magnetic field input data are normalized to the range of [−1, 1] to eliminate the magnitude differences between different magnetic field components and accelerate model convergence. The entire dataset is randomly partitioned into a training set and a test set with a fixed ratio of 2:1, where two-thirds of the samples are used for model training and the remaining one-third for performance testing. All three hidden layers adopt the tansig transfer function to enhance the nonlinear fitting capability for complex magnetic field-distance mapping, and the network adopts the trainlm training algorithm based on the Levenberg–Marquardt optimization combining Gauss–Newton and gradient descent. The training stopping criteria are defined as follows: the initial learning rate is 0.01; the training error goal is defined as 10^−5^; the maximum validation failure count is set to 50; and the maximum training epochs is configured to an extremely large value to ensure the model terminates training preferentially based on the error convergence criterion.

**Figure 1:**
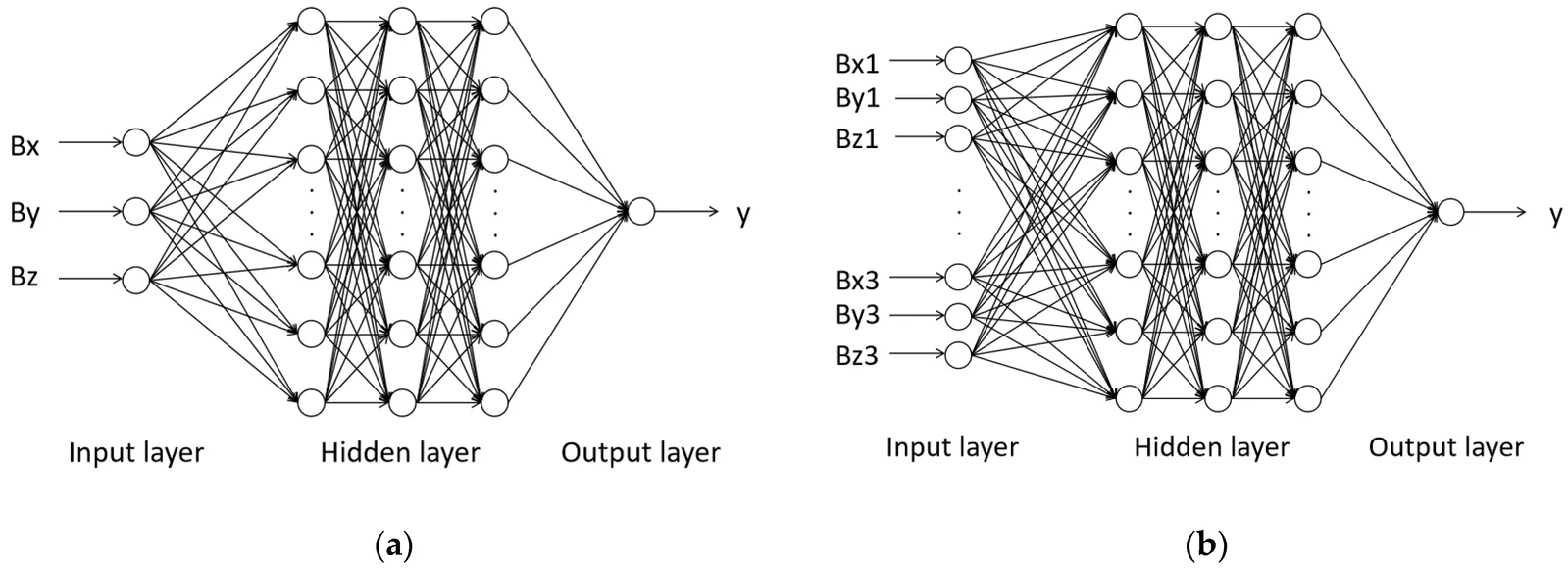
The structure of DNNs: (**a**) a single sensor; (**b**) three sensors.

Single-Sensor Configuration: As shown in Figure 1a, when a single sensor is used, the input vector is defined as X_t_ = [B_x_, B_y_, B_z_]^T^, where B_x_, B_y_, and B_z_ represent the magnetic flux density components measured along the *x*, *y*, and *z* axes, respectively. The output vector is Y_t_ = [y]^T^, where *y* denotes the distance inferred from the measured magnetic flux density. The training dataset consists of ranging data {(X_t_, Y_t_)|t = 1, 2, …, N}, where *N* denotes the total count of training samples.

Three-Sensor Configuration: As shown in Figure 1b, when three sensors are used, nine features are used as inputs for the DNN. These inputs are denoted as X_t_ = [B_x1_, B_y1_, B_z1_, B_x2_, B_y2_, B_z2_, B_x3_, B_y3_, B_z3_]^T^. The output vector is Y_t_ = [y]^T^, and the training dataset is denoted as {(X_t_, Y_t_)|t_3 = 1, 2, …, N}, where *N* denotes the total count of training samples.

The mathematical relationship between the input magnetic data and the output distance is formulated as

(1) $$\begin{array}{c} \sum_{k=1}^{l}O_{k}^{\prime }f\left(\sum_{j=1}^{n}H_{j}^{\prime }f\left(\sum_{i=1}^{m}V_{i}^{\prime }f\left(W_{i}^{\prime }X_{t}-\theta_{i}\right)-\theta_{j}\right)-\theta_{k}\right)=Y_{t}, \end{array}$$

where $W_{i}^{\prime }$ = [w_1i_, w_2i_, w_3i_]^T^ represents the connection weights between the input neurons and the *i*-th neuron in the first hidden layer, enabling initial nonlinear feature extraction from the original magnetic field signal. Moreover, $V_{i}^{\prime }$ = [v_i1_, v_i2_,..., v_in_]^T^ denotes the connection weights linking the *i*-th neuron of the first hidden layer to the second hidden layer, used to extract deep-level nonlinear features of magnetic field variations with distance. $H_{j}^{\prime }$ = [h_j1_, h_j2_,…, h_jl_]^T^ represents the weights connecting the *j*-th neuron in the second hidden layer to the third hidden layer, further fusing features to enhance distance resolution capability. $O_{k}^{\prime }$ = [O_k_]^T^ is the connection weight linking the *k*-th neuron of the third hidden layer to the output layer, ultimately mapping to the estimated distance. The biases are denoted as θ_i_, θ_j_, and θ_k_, which adjust the activation thresholds of the neurons. The activation function of the hidden layers is denoted as f(·), effectively fitting the complex nonlinear correlation between the magnetic field and distance.

These networks are capable of fitting complex magnetic fields and learning the correlation between the magnetic field strength and target distance, achieving high precision and computational efficiency in distance measurement for magnetic targets. To evaluate the accuracy of distance measurements, we quantify the distance error, defined as the deviation between the measured and actual distances. Specifically, this performance is evaluated using mean absolute error (MAE) and root mean square error (RMSE), which are mathematically formulated as follows:

(2) $$\begin{array}{c} \mathrm{MAE}=\frac{1}{n}\sum_{i=1}^{n}\left|a_{\mathrm{mi}}-a_{\mathrm{ei}}\right|, \end{array}$$

(3) $$\begin{array}{c} \mathrm{RMSE}=\sqrt{\frac{1}{n}\sum_{i=1}^{n}{\left(a_{\mathrm{mi}}-a_{\mathrm{ei}}\right)}^{2}}, \end{array}$$

where *n* represents the number of samples in the test set, *i* denotes a sample point in the test set, *a_mi_* represents the actual measured distance of the magnetic target, and *a_ei_* represents the distance of the magnetic target estimated by the DNN.

## 3. Simulation Experiments

This section details the simulation framework utilized to generate training datasets via Ansys finite element analysis. These datasets comprise magnetic field measurements collected over identical spatial ranges but with varying distance sampling step sizes. The primary objective is to evaluate how different spatial sampling resolutions impact the training performance of the deep neural network. Additionally, we comparatively analyze the predictive efficacy of models trained on data derived from a single-sensor baseline versus a three-sensor array.

### 3.1. Data Acquisition and Simulation Setup

The finite-element simulation was conducted using Ansys Maxwell 2021, with the magnetostatic solver adopted to calculate the static magnetic-field distribution generated by the permanent magnet [29]. The computational domain was divided into several regions with different mesh densities. A relatively coarse mesh was used in the outer air region, while a locally refined mesh was applied around the permanent magnet and the sensor positions to improve the accuracy of the extracted magnetic flux density. The maximum element sizes in the outer, middle, and locally refined regions were set to 0.4 mm, 0.2 mm, and 0.01 mm, respectively. The adaptive meshing process was terminated when the relative field-energy variation was below 1% or when the maximum number of adaptive passes reached 10.

The magnetic sensors were modeled as passive field-sampling probes located at the predefined sensor positions. At each magnet position, the three orthogonal magnetic flux density components, namely B_x_, B_y_, and B_z_, were extracted from the corresponding sensor coordinates. Therefore, the simulation does not include the electronic noise, offset, temperature drift, or finite bandwidth of the physical sensors. This idealized setting is mainly used to generate training data and to evaluate the fitting capability of the DNN under controlled magnetic-field conditions.

The cylindrical N52 NdFeB permanent magnet was selected because it is consistent with the physical magnet used in the experimental platform. Its finite geometry and axial magnetization are included in the finite-element model. Nevertheless, the proposed data-driven framework is not restricted to a specific magnet shape. When the training data are regenerated for a different magnet geometry, the DNN can learn the corresponding magnetic-field-to-distance mapping.

**Figure 2:**
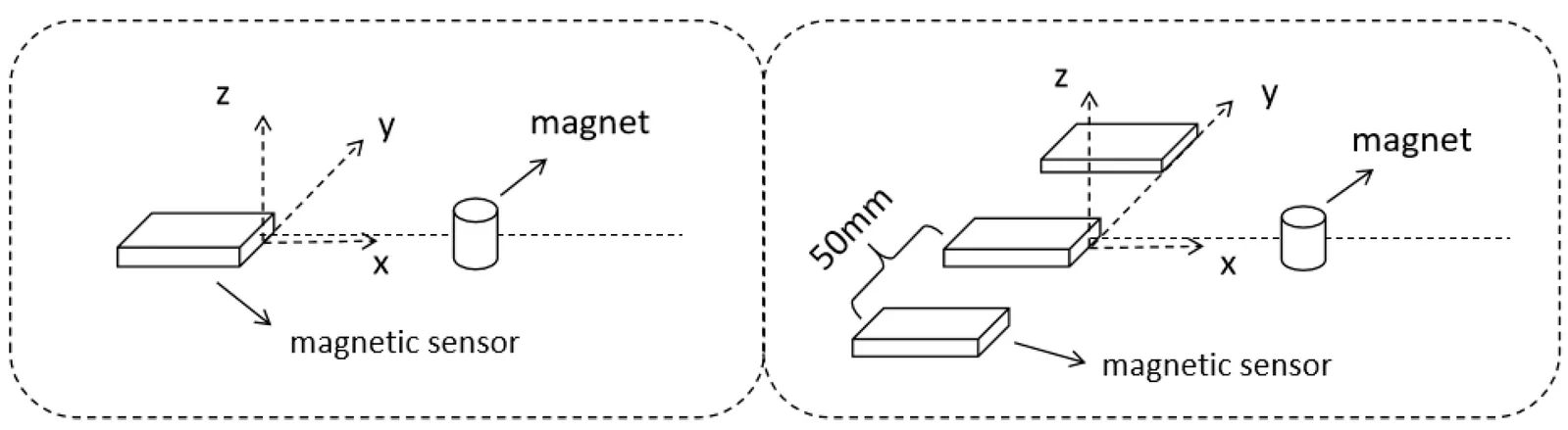
Schematic diagram of sensor and permanent magnet placement.

In this study, comparative distance measurement experiments were conducted using a DNN to process data from both a single sensor and a three-sensor array. As shown in Figure 2, a magnet moves along the x-axis from 0 to 140 mm. To maintain consistency with the subsequent physical experimental platform, the three sensors in the array are placed on the same plane and evenly spaced along the y-axis with a constant spacing of 50 mm between adjacent units.

For the three-sensor configuration, the spacing between adjacent sensors was set to 50 mm according to the mechanical layout of the experimental prototype. This spacing also provides distinguishable magnetic-field observations along the transverse direction. If the sensors are placed too close to each other, the measured magnetic-field components tend to be highly correlated, and the benefit of using multiple sensors becomes limited. If the sensors are placed too far apart, the magnetic field measured by some sensors may become weak, which may reduce the useful information for distance estimation.

It should be noted that the signals from the three sensors are not averaged during the measurement process. Instead, the three-axis magnetic-field components from all sensors are concatenated as a nine-dimensional input vector, i.e., X_t_ = [B_x1_, B_y1_, B_z1_, B_x2_, B_y2_, B_z2_, B_x3_, B_y3_, B_z3_]^T^.

The DNN then learns the nonlinear mapping from this multi-sensor magnetic-field vector to the target distance. In this way, the spatial diversity provided by the three sensors can be exploited by the network.

During the simulation, the permanent magnet moves along the x-axis from 0 to 140 mm at step sizes of 0.5 mm, 0.05 mm, and 0.005 mm. At each position, the three components (B_x_, B_y_, B_z_) of the magnetic flux density are collected, forming the three-axis magnetic dataset for training the deep neural network. To evaluate and compare training performance, the data from the single-sensor and three-sensor systems are trained separately.

**Figure 3:**
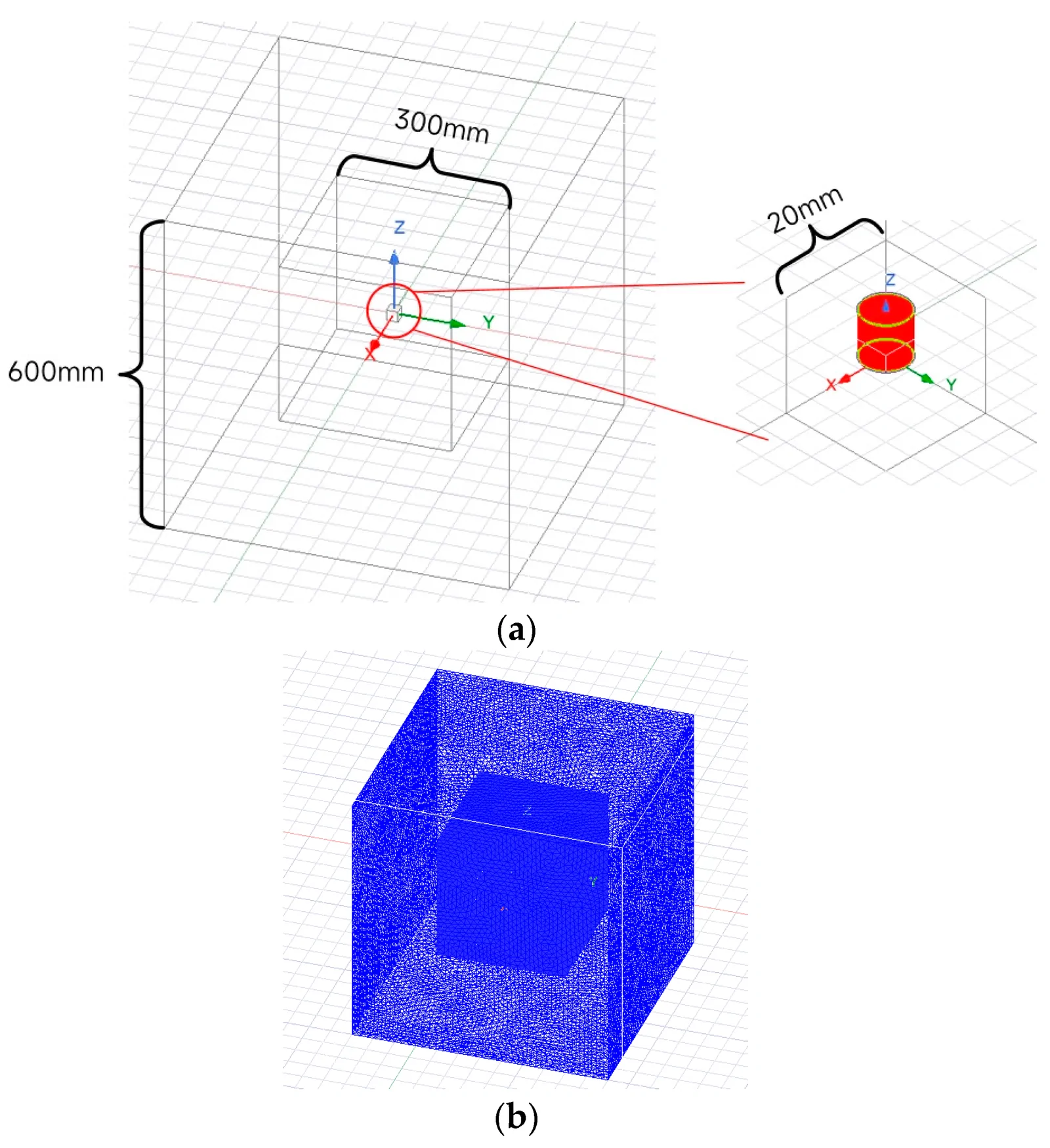
(**a**) Hierarchical mesh partitioning of the model during simulation preprocessing; (**b**) example of the model mesh after simulation.

The magnetic target is modeled as a cylindrical NdFeB permanent magnet of grade N52, with both diameter and height measuring 8 mm. The remanence Br is 1.45 T, the coercivity Hc is 860 KA/m, and the magnetization direction is axial. Given the expansive computational domain and the necessity for high precision, a hierarchical meshing strategy was implemented. As illustrated in Figure 3, the spatial domain is partitioned into three concentric layers, with the mesh density progressively refined toward the innermost region containing the magnet. The permanent magnet moves along the x-axis from 0 to 140 mm. Magnetic field data within this range are extracted from the simulation model.

**Figure 4:**
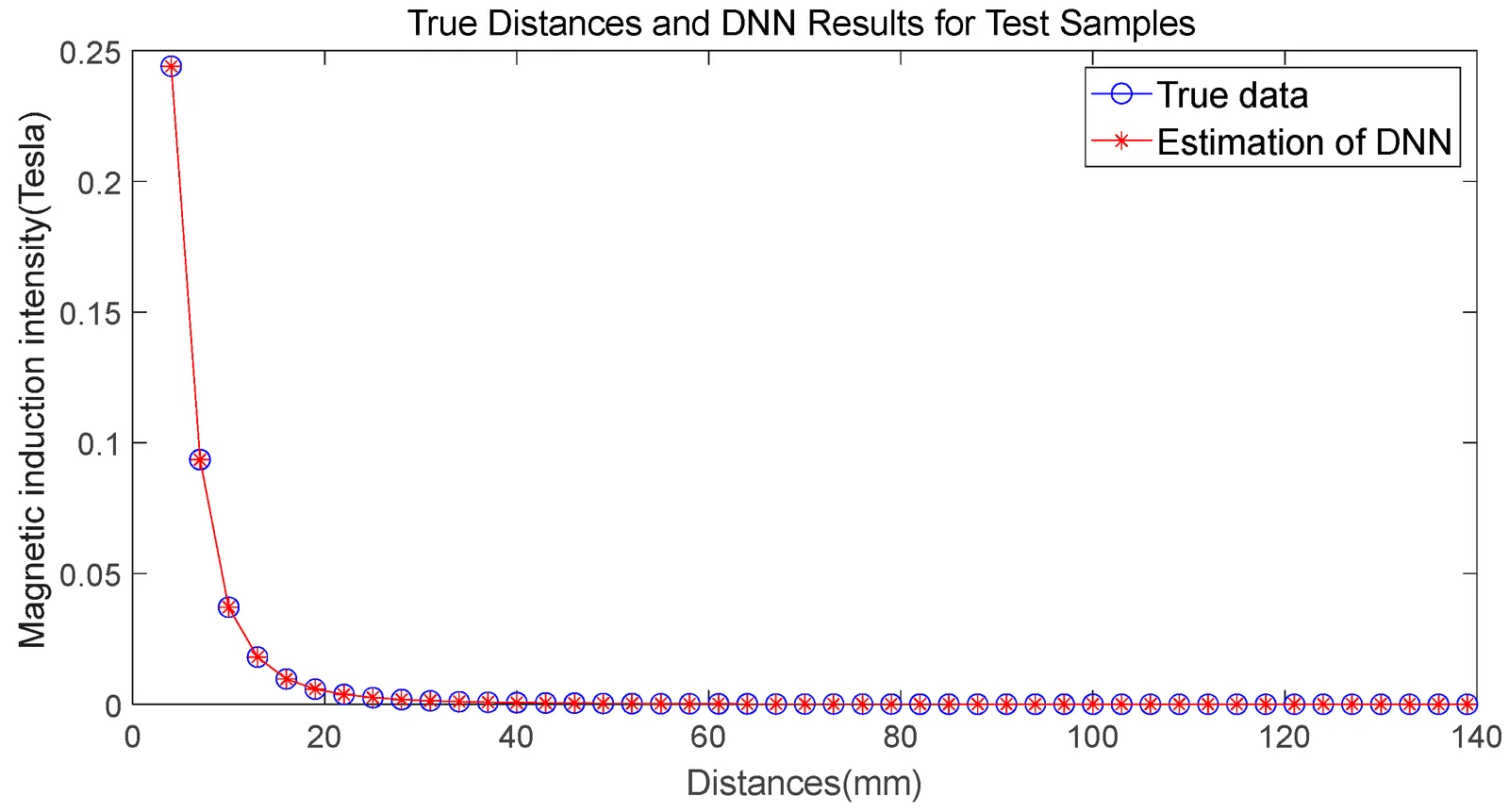
Magnetic field strength at each point along the line.

Through neural network training, the moving distance of the permanent magnet is mapped to the corresponding magnetic field strength. As shown in Figure 4, the horizontal axis represents the measured distance, while the vertical axis denotes the scalar value of the magnetic field intensity *Mag_B_*, which is given by

(4) $$\begin{array}{c} \mathrm{Ma}g_{B}=\sqrt{B_{x}^{2}+B_{y}^{2}+B_{z}^{2}}, \end{array}$$

where B_x_, B_y_, and B_z_ denote the orthogonal components of the magnetic flux density along the respective coordinate axes. Utilizing this scalar magnitude effectively encapsulates the overall spatial attenuation of the magnetic field as the relative distance increases.

### 3.2. Comparison of DNN Training Performance

**Figure 5:**
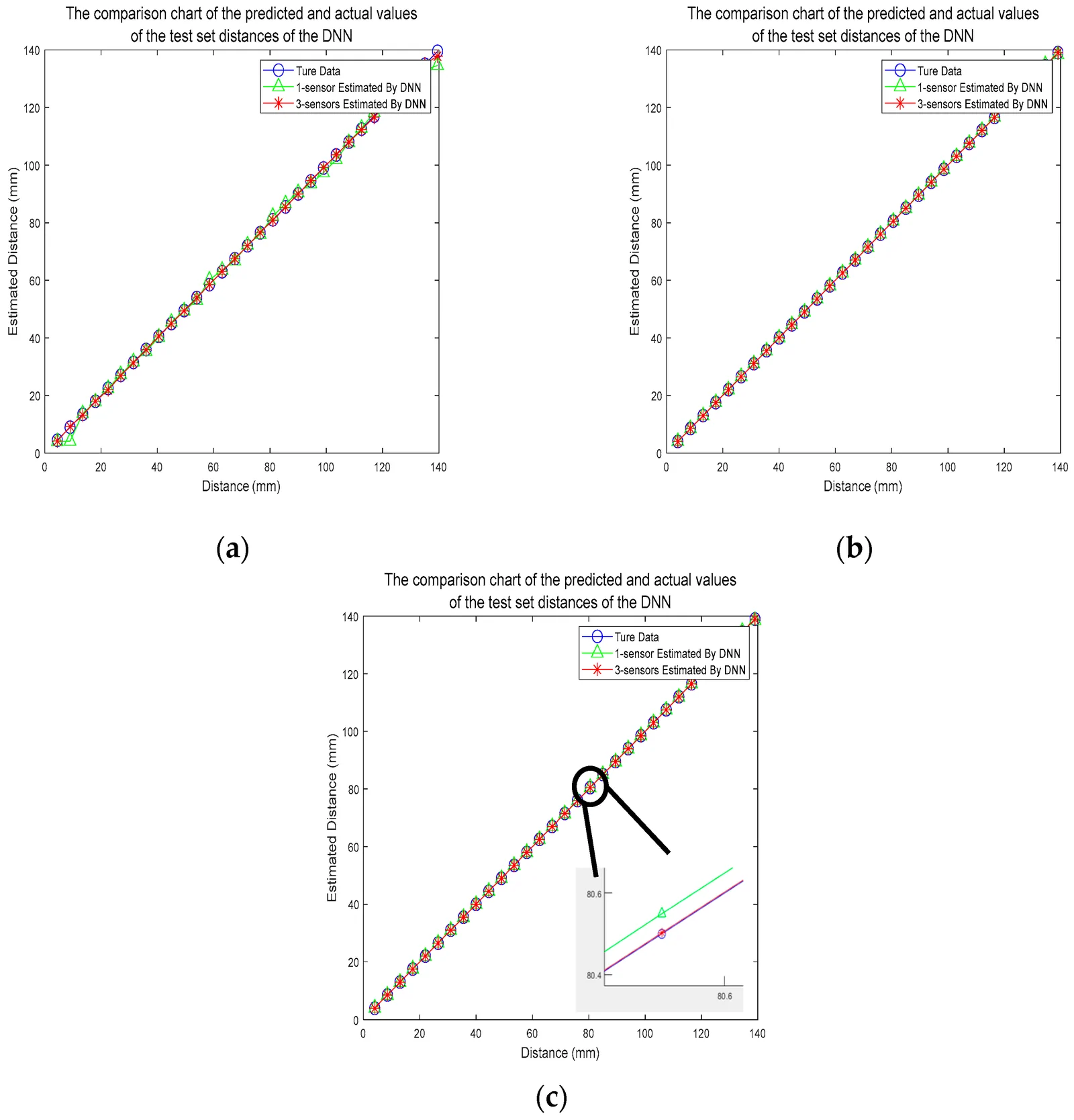
Measurement error results from the DNN: (**a**) distance sampling step of 0.5 mm, (**b**) distance sampling step of 0.05 mm, (**c**) distance sampling step of 0.005 mm.

A DNN is used to fit the relationship between magnetic field intensity and spatial position, causing the distance measured by the sensor to have a linear relationship with the actual distance. Figure 5 illustrates the training performance comparison of DNNs for distance measurements made by one sensor and three sensors under sampling steps of 0.5 mm, 0.05 mm, and 0.005 mm, respectively. The inset in Figure 5c shows a local magnification of a single point: the green triangle represents the predicted value from a single sensor, the red star denotes the predicted value from all three sensors, and the blue circle indicates the actual value.

**Figure 6:**
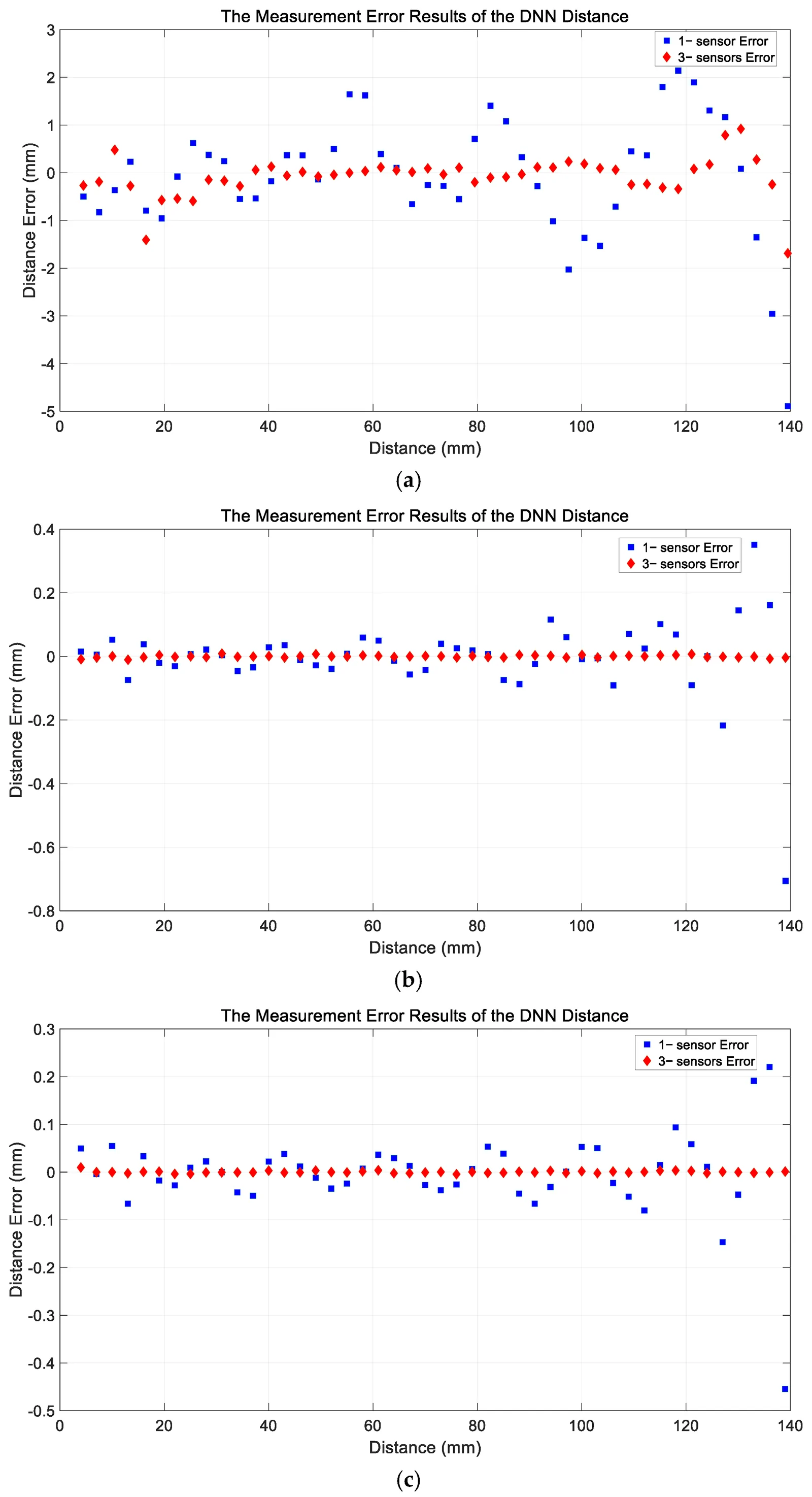
Comparison of errors between a single sensor and three sensors: (**a**) distance sampling step of 0.5 mm, (**b**) distance sampling step of 0.05 mm, (**c**) distance sampling step of 0.005 mm.

A comparison of the errors at each point is presented in Figure 6, where the measurement error results are those from Figure 5. At a sampling step size of 0.5 mm, the single sensor achieves an MAE of 0.9405 mm and an RMSE of 1.3 mm, while the three-sensor configuration achieves an MAE of 0.2407 mm and an RMSE of 0.3819 mm. At a step size of 0.05 mm, the single sensor yields an MAE of 0.0641 mm and an RMSE of 0.1247 mm, whereas the three-sensor setup gives an MAE of 0.0031 mm and an RMSE of 0.0043 mm. At a step size of 0.005 mm, the single sensor achieves an MAE of 0.0503 mm and an RMSE of 0.0909 mm, while the three-sensor configuration achieves an MAE of 0.0016 mm and an RMSE of 0.0023 mm. In terms of sampling step size, a smaller distance step results in a smaller error. With regard to the number of sensors, the predicted values from the three sensors are nearer to the actual values and yield smaller errors compared to those from the single sensor.

Table 1 and Table 2 provide detailed listings of the MAE and RMSE for different sampling step sizes using a single sensor and three sensors, respectively.

Table 1 and Table 2 show that single-sensor magnetic field data exhibit low informational dimensionality and weak robustness, making the model susceptible to noise interference and limiting its learning capacity. In contrast, the three-sensor configuration provides complementary high-dimensional information, enhancing the anti-interference capability of the extracted features. With a large sampling step size, the system leverages spatial distribution characteristics and information redundancy to compensate for data scarcity, stabilizing the model’s mapping relationship. With a small sampling step size, the multi-sensor configuration fully exploits multi-source information to construct a more accurate “distance–magnetic field intensity” mapping model, achieving generalization performance far superior to the single-sensor approach. While increasing the sample size improves model performance, this improvement exhibits diminishing returns as the sample count grows.

## 4. Experiments and Analysis

**Figure 7:**
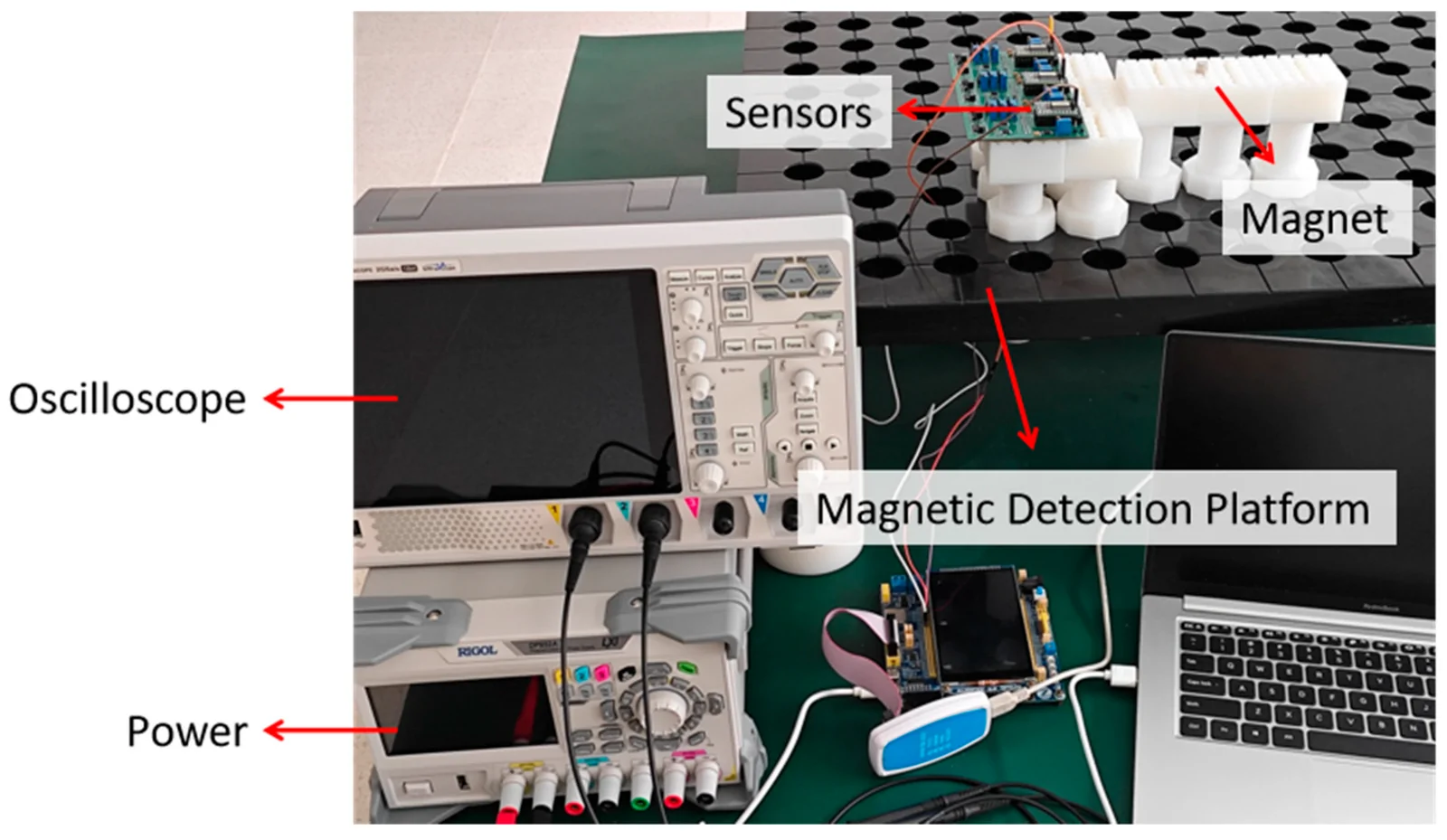
Experimental platform.

While the previous section focused on computational simulations, this section presents the construction of a physical experimental platform designed for empirical validation. The objective is to evaluate the trained DNN models using magnetic sensor data and to analyze the resulting measurement errors under practical conditions. The architecture of the experimental platform is presented in Figure 7.

**Figure 8:**
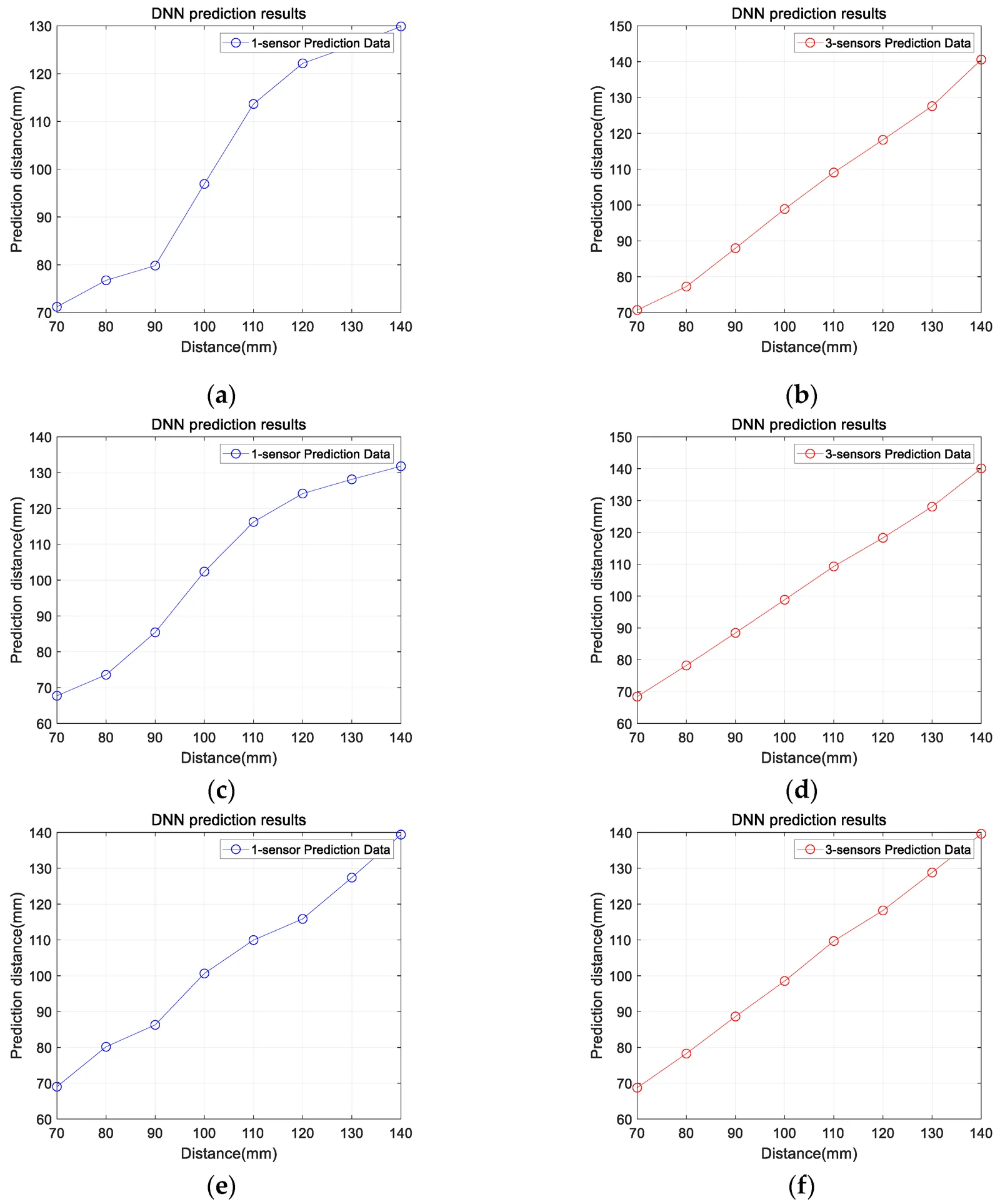
DNN prediction results: (**a**,**b**) distance predictions for a single sensor and for three sensors, with a sampling step size of 0.5 mm; (**c**,**d**) distance predictions for a single sensor and for three sensors, with a sampling step size of 0.05 mm; (**e**,**f**) distance predictions for a single sensor and for three sensors, with a sampling step size of 0.005 mm.

In the experimental setup, the magnet is moved in the range of 70–140 mm relative to the sensor. Due to the mechanical structure and fixed positioning slots of the prototype platform, eight discrete test positions are available within this range. Therefore, the experimental results are used as a prototype-level validation of the trained DNN model rather than a fully dense spatial generalization test. During the experimental process, magnetic field data were collected via the sensors and fed into the previously trained neural network to predict measurement errors. The resulting errors are presented in Figure 8 and Table 3 and Table 4.

At each available test position, repeated measurements were conducted to reduce the influence of random fluctuations, and the averaged prediction errors were used for performance evaluation. The three-sensor system demonstrates superior distance measurement performance, with its prediction curves exhibiting greater linearity than those of the single-sensor configuration. As shown in Figure 8a,c,e for the single-sensor setup and Figure 8b,d,f for the three-sensor system, the prediction curves of the three-sensor system are notably more linear.

Table 3 and Table 4 summarize the error for the eight sampling points within the 70–140 mm range, derived from the neural network prediction results presented in Figure 8.

To further quantify the linearity of the experimental prediction results, the predicted distance in Figure 8 was fitted using a linear function, namely

(5) $$\begin{array}{c} \hat{d}=\mathrm{kd}+b, \end{array}$$

where *d* denotes the actual distance (mm), $\hat{d}$ denotes the DNN-predicted distance, *k* is the fitted slope, and *b* is the fitted intercept. The coefficient of determination *R*^2^, which quantifies the goodness of fit, is defined as

(6) $$\begin{array}{c} R^{2}=1-\frac{\sum_{i=1}^{n}{\left(d_{i}-{\hat{d}}_{i}\right)}^{2}}{\sum_{i=1}^{n}{\left(d_{i}-\bar{d}\right)}^{2}}, \end{array}$$

where *d_i_* is the corresponding true measured distance of the *i*-th sample, ${\hat{d}}_{i}$ the DNN-predicted distance of the *i*-th sample, $\bar{d}$ represents the average value of the measured actual distances, and *n* is the total number of sample points. For an ideal prediction result, *k*, *b*, and *R*^2^ should approach 1, 0, and 1, respectively. The extracted values of these parameters under different experimental conditions are summarized in Table 3.

The fitting results in Table 5 show that the prediction curves obtained from the three-sensor configuration are closer to the ideal linear relationship than those obtained from the single-sensor configuration. This indicates that the use of multiple sensors improves the stability and linearity of the DNN prediction results by providing complementary spatial magnetic-field information.

To further analyze the discrepancy between the simulation and experimental results, Table 6 summarizes the absolute error gap between them under the same sensor configurations and sampling step sizes. It should be noted that the simulation results are obtained under ideal finite-element conditions, whereas the experimental results are collected using the prototype platform with unavoidable hardware and environmental disturbances.

The deviations of mean absolute error (MAE) and root mean square error (RMSE) between simulation and experimental results are defined as

(7) $$\begin{array}{c} ∆\mathrm{MAE}=\left|{\mathrm{MAE}}_{\exp}-{\mathrm{MAE}}_{\mathrm{sim}}\right|, \end{array}$$

(8) $$\begin{array}{c} ∆\mathrm{RMSE}=\left|{\mathrm{RMSE}}_{\exp}-{\mathrm{RMSE}}_{\mathrm{sim}}\right|, \end{array}$$

where the subscripts *exp* and *sim* represent experimental and simulated results, respectively. The quantitative comparison results under different sensor configurations and sampling step sizes are listed in Table 6.

As for existing magnetic positioning methods, Su et al. [24] proposed the few-shot learning network AMagPoseNet for magnet pose estimation, with a localization error of 1.87 ± 1.14 mm, whereas the present study demonstrates a notable advantage even when using a single sensor with a sampling step size of 0.005 mm, achieving an error of 1.6065 mm. Furthermore, it is clearly indicated in Table 5 that the error is significantly reduced to approximately 1.1881 mm when three sensors are employed.

Compared with our earlier work [26], which focused on verifying the applicability of neural networks in magnetic measurement and optimizing model performance under fixed settings, the current work centers on two pivotal factors, i.e., sampling step size and sensor quantity. Accordingly, we quantitatively analyze their effects on the network prediction accuracy and summarize the error characteristics through simulations and practical tests.

As shown in Table 6, the experimental errors are consistently larger than the simulation errors. This gap is mainly caused by the difference between the ideal simulation environment and the practical measurement platform. In the finite-element simulation, the magnet geometry, magnetization direction, sensor positions, and magnetic field distribution are all precisely defined, and the data are free from environmental magnetic interference and acquisition noise. In contrast, the experimental platform inevitably introduces sensor noise, sensor offset, mechanical positioning tolerance, alignment error between the magnet and sensors, data acquisition resolution limits, and background magnetic disturbances.

In addition, the magnetic field strength decreases rapidly as the distance increases. In the 70–140 mm experimental range, the magnetic field gradient becomes relatively small. Therefore, a small fluctuation in the measured magnetic field may result in a noticeable distance prediction error. This effect can be qualitatively described by

(9) $$\begin{array}{c} ∆d\approx \frac{\left|∆B\right|}{\left|\partial B∕\partial d\right|}, \end{array}$$

where ∆*B* is the fluctuation of the measured magnetic field, and *∂B*/*∂d* denotes the magnetic field gradient with respect to distance. A smaller value of |*∂B*/*∂d*| indicates that the same magnetic field noise will produce a larger prediction error in distance.

## 5. Conclusions

This paper investigates the influence of both different distance sampling step sizes and the number of deployed sensors on the prediction accuracy of deep neural networks. In this study, a dataset with correspondence between distance and magnetic field strength was constructed through simulation to train a DNN. Subsequently, magnetic data acquired from a physical experimental platform were employed to validate the model’s predictive performance in practical scenarios. The simulation results indicate that the spatial sampling resolution significantly affects the precision of the DNN model. For a single-sensor setup, reducing the distance sampling step from 0.5 mm to 0.005 mm decreased the MAE from 0.9405 mm to 0.0503 mm. Under the same conditions, the three-sensor configuration further reduced the MAE to 0.0016 mm. These results suggest that finer sampling steps and sensor redundancy contribute to more stable mapping and higher theoretical accuracy.

Physical experimental evaluations conducted within a 70–140 mm range were consistent with the simulation trends. With a 0.005 mm sampling step, the three-sensor system achieved an MAE of 1.1881 mm, outperforming the single-sensor baseline of 1.6065 mm. The multi-sensor approach also demonstrated better output linearity and robustness against environmental noise. In summary, optimizing the distance sampling step size and employing a multi-sensor architecture can effectively refine the accuracy and reliability of magnetic distance measurement systems.

## Figures and Tables

**Table 1:** Comparison of DNN training performance using different sampling step sizes with a single sensor.

Sampling Step Size (mm)	MAE (mm)	RMSE (mm)	Error Range (mm)
0.5	0.9405	1.3	−4.9–2.1
0.05	0.0641	0.1247	−1.2–0.3607
0.005	0.0503	0.0909	−0.9978–0.2489

**Table 2:** Comparison of DNN training performance using different sampling step sizes with three sensors.

Sampling Step Size (mm)	MAE (mm)	RMSE (mm)	Error Range (mm)
0.5	0.2407	0.3819	−1.7–0.9195
0.05	0.0031	0.0043	−0.0388–0.0209
0.005	0.0016	0.0023	−0.0108–0.0119

**Table 3:** Position error of the permanent magnet when using a single sensor.

Sampling Step Size (mm)	MAE (mm)	RMSE (mm)
0.5	4.7256	5.7313
0.05	3.5153	4.0003
0.005	1.6065	2.2178

**Table 4:** Position error of the permanent magnet when using three sensors.

Sampling Step Size (mm)	MAE (mm)	RMSE (mm)
0.5	1.5404	1.7210
0.05	1.2144	1.3341
0.005	1.1881	1.2969

**Table 5:** Linear fitting results of the experimental DNN predictions in Figure 8.

Sensor Configuration	Sampling Step Size (mm)	Slope *k*	Intercept *b* (mm)	*R* ^2^
Single sensor	0.5	0.9523	2.0316	0.9543
Single sensor	0.05	1.0134	−2.7229	0.9588
Single sensor	0.005	0.9841	0.2647	0.9945
Three sensors	0.5	1.0024	−1.4656	0.9972
Three sensors	0.05	1.0128	−2.6430	0.9994
Three sensors	0.005	1.0102	−2.2571	0.9996

**Table 6:** Quantitative comparison between simulation and experimental errors.

Sensor Configuration	Sampling Step Size (mm)	∆MAE (mm)	∆RMSE (mm)
Single sensor	0.5	3.7851	4.4313
Single sensor	0.05	3.4512	3.4512
Single sensor	0.005	1.5562	2.1269
Three sensors	0.5	1.2997	1.3391
Three sensors	0.05	1.2113	1.3298
Three sensors	0.005	1.1865	1.2946

## Data Availability

The data presented in this study are available upon request from the corresponding author.

## References

[1] Yeh S.C., Hsu W.-H., Lin W.-Y., Wu Y.-F. (2019). Study on an indoor positioning system using Earth’s magnetic field. IEEE Trans. Instrum. Meas..

[2] Fu Q., Fan C., Wang X., Zhang S., Zhang X., Guo J., Guo S. (2021). A compensation method for magnetic localization on capsule robot in medical application. IEEE Sens. J..

[3] Hu C., Li M., Song S., Yang W., Zhang R., Meng M.Q.-H. (2010). A cubic 3-axis magnetic sensor array for wirelessly tracking magnet position and orientation. IEEE Sens. J..

[4] Allen G.I., Sulzberger G., Bono J.T., Pray J.S., Clem T.R. (2005). Initial Evaluation of the New Real-time Tracking Gradiometer Designed for Small Unmanned Underwater Vehicles. Proceedings of OCEANS 2005 MTS/IEEE.

[5] Park D., Kwak K., Chung W.K., Kim J. (2015). Development of underwater short-range sensor using electromagnetic wave attenuation. IEEE J. Ocean. Eng..

[6] Brossard M., Bonnabel S., Barrau A. (2020). Denoising IMU gyroscopes with deep learning for open-loop attitude estimation. IEEE Robot. Autom. Lett..

[7] Sherman J.T., Lubkert J.K., Popovic R.S., DiSilvestro M.R. (2007). Characterization of a novel magnetic tracking system. IEEE Trans. Magn..

[8] Schlageter V., Besse P.A., Popovic R.S., Kucera P. (2001). Tracking system with five degrees of freedom using a 2D-array of Hall sensors and a permanent magnet. Sens. Actuators A Phys..

[9] Taghvaeeyan S., Rajamani R. (2015). Magnetic sensor-based large distance position estimation with disturbance compensation. IEEE Sens. J..

[10] Gao J., Jiang Z., Li B., Shen Y., Wang W., Dong H., Wang J., Pan L., Chen J. (2024). Low-frequency environmental magnetic noise elimination based on a neural network algorithm for TMR sensor arrays. IEEE Sens. J..

[11] Meier P., Rohrmann K., Sandner M., Prochaska M., Ferhi O. (2023). On the treatment of measurement errors for magnetic angular sensors with neural networks. IEEE Sens. J..

[12] Stefanoni M., Takács M., Odry Á., Sarcevic P. (2025). A Comparison of Neural Networks and Fuzzy Inference Systems for the Identification of Magnetic Disturbances in Mobile Robot Localization. Acta Polytech. Hung..

[13] Huang Y., Liu Z., Zhao Y., Zhu Y., Liu J., Wang S. (2025). Innovative Magnetic Localization for Close-Range Scenarios: Bi-GRU With Adaptive Sensor Selection and Spatial Feature Extraction. IEEE Sens. J..

[14] Wang Z., Lu J., Liu Z., Li X., Sheng J., Li J. (2025). Neural network assisted magnetic moment measurement using an atomic magnetometer. IEEE Trans. Instrum. Meas..

[15] Licciardi S., Ala G., Francomano E., Viola F., Giudice M.L., Salvini A., Sargeni F., Bertolini V., Di Schino A., Faba A. (2024). Neural network architectures and magnetic hysteresis: Overview and comparisons. Mathematics.

[16] Lv B., Chen Y., Dai H., Su S., Lin M. (2019). PKBPNN-based tracking range extending approach for TMR magnetic tracking system. IEEE Access.

[17] Wen T., Liu S., Wang H. (2024). Soft-Magnet-Based Position Estimation Using an Approximate Extended Kalman Filter With a Hybrid Analytical-Neural Network Model. IEEE Trans. Instrum. Meas..

[18] Ribeiro P., Neto M., Cardoso S. (2018). Strategy for determining a magnet position in a 2-D space using 1-D sensors. IEEE Trans. Magn..

[19] Fan L., Hu H., Zhang X., Wang H., Kang C. (2022). Magnetic anomaly detection using one-dimensional convolutional neural network with multi-feature fusion. IEEE Sens. J..

[20] Hu M., Jing S., Du C., Xia M., Peng X., Guo H. (2020). Magnetic dipole target signal detection via convolutional neural network. IEEE Geosci. Remote Sens. Lett..

[21] You X., Zhang J., Chen B., Zhang K., Liu X., Yan B., Zhu W. (2025). Magnetic Moment Estimation Algorithm Based on Convolutional Neural Network. Appl. Sci..

[22] Belalcazar-Bolaños E.A., Torricelli D., Pons J.L. (2023). Automatic detection of magnetic disturbances in magnetic inertial measurement unit sensors based on recurrent neural networks. Sensors.

[23] Chen S., Zhu M., Zhang Q., Cai X., Bo X. (2019). Accurate Magnetic Object Localization Using Artificial Neural Network. 2019 15th International Conference on Mobile Ad-Hoc and Sensor Networks (MSN).

[24] Su S., Yuan S., Xu M., Gao H., Yang X., Ren H. (2023). AMagPoseNet: Real-time six-DoF magnet pose estimation by dual-domain few-shot learning from prior model. IEEE Trans. Ind. Inform..

[25] Wang Y., Pittiglio G., Lin X., Dupont P.E. (2025). Few-Shot Magnet Localization Using Sim-to-Real Transfer Learning. IEEE Robot. Autom. Lett..

[26] Qiu C., Qian Z., Qi Q., Wang R., Li X., Bai R. (2025). AI-Assisted Passive Magnetic Distance/Position Sensor. Sensors.

[27] Ni Y., Sun Y., Zhang H., Li X., Zhang S., Li M. (2023). Data-Driven navigation of ferromagnetic soft continuum robots based on machine learning. Adv. Intell. Syst..

[28] Zhang X., Liu Z., Wu T., Wang B. (2025). Corotational dynamic modeling and PSO-DNN framework for hard-magnetic soft elastomers. Int. J. Mech. Sci..

[29] Li H., Liu F. (2011). The electromagnetic field analysis of permanent magnet synchronous generator based on ANSYS. Adv. Mater. Res..

